# Can nutrition education mitigate the impacts of COVID‐19 on dietary quality? Cluster‐randomised controlled trial evidence in Myanmar's Central Dry Zone

**DOI:** 10.1111/mcn.13259

**Published:** 2021-08-19

**Authors:** Catherine Ragasa, Isabel Lambrecht, Kristi Mahrt, Hongdi Zhao, Zin Wai Aung, Jessica Scott

**Affiliations:** ^1^ Development Strategy and Governance Division (DSGD) International Food Policy Research Institute (IFPRI) Washington District of Columbia USA; ^2^ Development Strategy and Governance Division (DSGD) International Food Policy Research Institute (IFPRI) Yangon Myanmar; ^3^ Development Strategy and Governance Division (DSGD) International Food Policy Research Institute (IFPRI) Denver Colorado USA; ^4^ Development Strategy and Governance Division (DSGD) International Food Policy Research Institute (IFPRI) Nay Pyi Taw Myanmar; ^5^ Gender and Nutrition, WorldFish Yangon Myanmar

**Keywords:** behaviour change communication, COVID‐19, dietary quality, impact evaluation, resilience

## Abstract

We evaluate the immediate impact of a nutrition and gender behaviour change communication on dietary quality in rural communities in Myanmar and assess whether the communication helped mitigate the effect of the COVID‐19 crisis on dietary quality. The intervention was designed and implemented as a cluster‐randomised controlled trial in which 15 villages received the intervention and 15 control villages did not. The intervention was implemented from June to October 2020. This paper provides an assessment of the intervention's impact on dietary quality based on the results of two phone surveys conducted in August and October 2020. Immediate impacts of the intervention indicate an improvement in women's dietary diversity scores by half a food group out of 10. At baseline, 44% of women were likely to have consumed inadequately diverse diets; results indicate that 6% (*p*‐value: 0.003, SE: 0.02) fewer sample women were likely to have consumed inadequately diverse diets. More women in treatment villages consumed pulses, nuts, eggs and Vitamin A‐rich foods daily than in control villages. In response to economic shocks related to COVID‐19, households in the treatment villages were less likely to reduce the quantity of meat and fish consumption than in control villages. The long‐term impacts of the intervention need to be continuously evaluated.

**Key messages**

- Adding simple nutrition and gender messaging to social protection programmes during COVID‐19 can improve dietary quality among women, especially in areas where health and nutrition practices and dietary diversity are low at baseline.
- Immediate impacts of the behavioural change communication indicate an improvement in women's dietary diversity scores by half a food group out of 10. Results indicate that 6% fewer sample women were likely to have consumed inadequately diverse diets.
- Further evaluation is needed to determine whether the immediate impacts are sustained over time.

## 1 INTRODUCTION

Since the onset of the COVID‐19 pandemic, researchers have highlighted the threats that income shocks and disruptions in agri‐food systems pose to poverty and food security, particularly among vulnerable groups (Headey, Heidkamp, et al., 2020; Headey & Ruel, 2020; Laborde et al., 2020; Torero, 2020). From the supply side, substantial disruptions in agriculture and food systems will hinder access to inputs and services, reduce production and potentially increase food prices. Border restrictions, lockdowns and outbreaks are slowing harvests, constraining food transport, forcing market closures and resulting in food loss (Aldaco et al., 2020; Amicarelli & Bux, 2020; IFPRI, 2020; Resnick, 2020). From a consumer perspective, reduced income may lead to less purchasing power for food, particularly among the vulnerable. It is estimated that, by the end of 2020, COVID‐19 resulted in an additional 130 million people becoming food insecure (Anthem, 2020); 45 million people becoming acutely food insecure, 33 million of whom live in South and Southeast Asia (UNSDG, 2020) and 6.7 million children younger than 5 years wasted and stunted (Headey, Heidkamp, et al., 2020).

Before the COVID‐19 crisis, Myanmar had achieved economic growth and development over the last decade. The proportion of the population living in poverty halved from 48.2% in 2005 to 24.8% in 2017 (CSO, UNDP, & World Bank, 2019). This progress is feared to have been substantially set back by the pandemic. Myanmar's first round of lockdown and other measures implemented in April 2020 were initially able to control the spread of the infection, but the crisis led to a sharp spike in poverty followed by a modest economic recovery in mid‐2020 (Diao & Mahrt, 2020; Headey, Heidkamp, et al., 2020).

A second, far worse phase of the crisis began in August 2020, accompanied by the imposition of a second lockdown and other measures in mid‐September 2020. Results from a high‐frequency International Food Policy Research Institute (IFPRI) survey show that Myanmar's second wave of COVID‐19 infections led to severe reductions in income, resulting in deeply worrying increases in poverty and food insecurity (Headey, Zaw Oo, et al., 2020). In a national household survey, 83% of households reported lower than usual income between March and October 2020, with an average decline in income of 43%; poor and vulnerable households were more likely than those that were financially secure to experience reductions in income (CSO, MoPFI, & UNDP, 2020).

As of January 2021, in a country with a population of 53.7 million, the number of people with insufficient food consumption increased to 15 million, with an increase of 0.37 million from October to December 2020 (WFP, 2021). Due to a lack of money, 30.7% and 40.5% of households urban and rural households, respectively, reported eating less than usual between March and October (CSO, MoPFI, & UNDP, 2020). Healthier diets cost more than unhealthy diets; as household income declines, it is likely for diets to shift toward cheaper, more energy‐dense, less micronutrient‐dense foods (Alemu et al., 2019; Green et al., 2013; Miller et al., 2016). In a sample of women with young children in Myanmar's Central Dry Zone, Maffioli et al. (2021) find that those with incomes affected by COVID‐19 experienced significantly lower food security and diet diversity.

Social protection programmes, particularly cash transfers, have become common strategies to mitigate the negative effects of the COVID‐19 crisis. However, studies have also shown that nutrition communication with and without cash transfers is effective in improving diets and nutritional status (Dewey & Adu‐Afarwuah, 2008). While behaviour change communication (BCC) interventions are recognised to be effective with additional material inputs or financial resources (Ferré & Sharif, 2014; Hoddinott & Skoufias, 2004; Lagarde et al., 2007; Zhang et al., 2018), research shows that intensive and well‐designed interventions without input or financial support, even in households that have challenges with affording food, can also lead to better dietary quality and other nutrition indicators (Frongillo et al., 2019; Murendo et al., 2018; Reinbott et al., 2016; Warren et al., 2020). More research is needed to evaluate nutrition‐only interventions and should include assessment of specific topics, BCC format, intervention context, strength of service delivery systems, recipient characteristics and length of exposure (Warren et al., 2020).

In Myanmar's Central Dry Zone, Maffioli et al. (2021) assess the long‐term impact of the Maternal and Child Cash Transfer (MCCT) Programme, which distributed cash transfers to pregnant women and those who had recently given birth. A subset of the recipients received the cash transfer coupled with BCC targeting dietary diversity and nutrition. Although the programme ended almost 2 years before the onset of COVID‐19, the authors find a sustained positive impact of the BCC intervention on maternal dietary diversity. Women provided with the nutrition BCC during the 2016–2019 programme period had higher dietary diversity in June–November 2020 than those not provided with the nutrition BCC.

This paper is among the first to evaluate an intervention that can mitigate the negative effects of COVID‐19 on dietary quality in rural communities. In particular, the study aims to test whether a BCC on nutrition and women's empowerment improves dietary quality during COVID‐19 in two rural areas in Myanmar's Central Dry Zone. This study was initially intended to provide insights on how to enhance crop diversification, dietary diversity and gender equality for future integration and scaling up in agricultural and irrigation projects. The emergence of the COVID‐19 crisis allows the study to also provide insights on the effectiveness of the BCC as a mitigation strategy to maintain or improve dietary quality in rural areas in the face of crisis. Moreover, though this study was originally planned without the use of transfers, the Government of Myanmar (GoM) widely expanded social protection cash transfers under the COVID‐19 Economic Recovery Plan. 1 This paper presents a midterm assessment of the nutrition and gender intervention implemented in June through October 2020 in randomly selected rural communities and its causal impact on dietary quality.

## 2 METHODS

### 2.1 Study site

**FIGURE 1:**
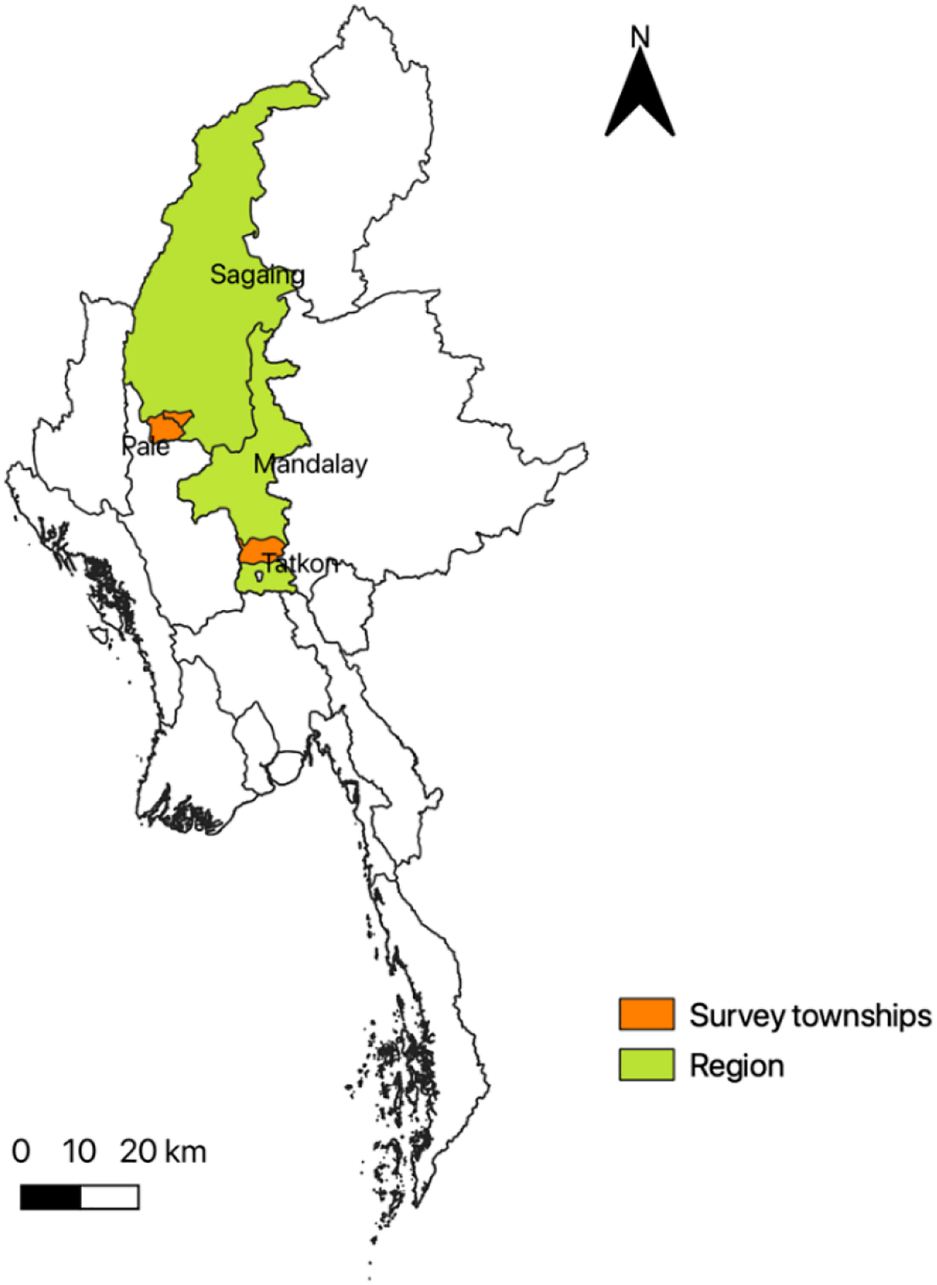
Map of Myanmar and location of the study sites

Myanmar's Central Dry Zone, an area with an estimated 12 million people or about 23% of Myanmar's total population, covers roughly a third of the country's grain cropping area (Herridge et al., 2019). This study focuses on 30 rural communities in the Central Dry Zone, which are located in the catchment areas of two irrigation sites: (1) Sinthe in Tatkone township in the Nay Pyi Taw region and (2) North Yamar in Pale and Yinmabin townships in the Sagaing region (Figure 1). Rice is primarily grown in the irrigation sites, and other crops such as legumes and oilseeds are grown in nonirrigation (upland) areas. Growing rainfed crops has high risks, and access to water for irrigation is critical to reduce risks as well as to expand farmers' options (Boutry et al., 2017). Households in the sample communities are relatively better‐off in terms of dwelling indicators compared to the average in the Central Dry Zone. This may be associated with greater economic opportunities coming from irrigation water access, relatively good infrastructure and access to markets. Most households have access to electricity and roads and are roughly 4–5 km or 20–30 min by motorbike from the nearest town centre, where household members might work in wage or salary employment or sell their produce. The main sources of purchased foods are vendors who come to the villages.

### 2.2 Intervention

**FIGURE 2:**
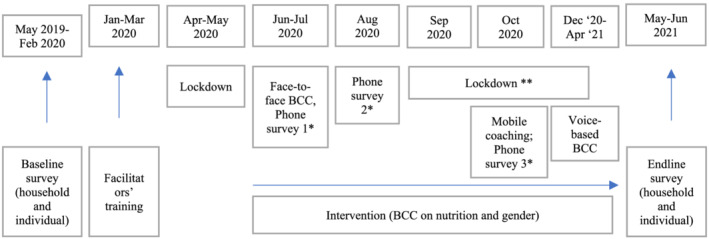
Time frame of the study. Source: Authors' illustration. Note: *Phone survey 1 asks respondents' experiences from February to May 2020 (onset of the pandemic), Phone survey 2 asks about experiences in June–July 2020 and Phone survey 3 asks about experiences in August–September 2020. The food group recall for measurement of dietary diversity pertains to the day before the interview. See also Table S1 for the details of the phone survey periods. **Lockdown and mobility restrictions are ongoing as of the writing of this paper

The intervention was a BCC on gender and nutrition implemented in June, July and October 2020 (Figure 2). To minimise the risk of contamination, we adopted a cluster‐randomised controlled trial in which the 30 relatively homogeneous sample villages are the clusters. We anticipate minimal differences across villages, strong information spillover within each village and minimal spillover across villages, justifying clustering and randomisation at the village level. We used a computer programme (Stata) to select villages and to randomly assign 15 villages into the treatment group (those receiving the BCC) and 15 villages into the control group (those not receiving the BCC). In each village, 29–32 households were randomly selected for the survey. In total, at baseline, there were 453 treatment households (those receiving BCC) and 465 control households (those not receiving BCC).

The Myanmar Institute of Gender Studies (MIGS) managed and implemented the BCC with the assistance of Save the Children and its partners who helped train nutrition facilitators. Nutrition messaging was designed around materials developed by Leveraging Essential Nutrition Actions to Reduce Malnutrition (LEARN). 2 LEARN's modules are designed specifically for the Myanmar context and cover nutrition basics, family nutrition, access to nutritious foods, food preparation and diet‐related taboos (LEARN, 2015). The complementary gender component of the intervention is a potential pathway for improving dietary quality by shifting aspects of intrahousehold dynamics between women and men; those aspects include division of work and decision‐making, particularly food decisions related to purchases, preparation and allocation (Ahmed et al., 2018; Farnworth et al., 2015). The study also aims to measure and track the impact of gender messaging on women's empowerment indicators to be measured during the endline evaluation survey. The gender messaging addresses gender and nutrition, as well as gender equality and equity, gender socialisation, gender stereotyping, gender‐based violence, sex and gender, power and patriarchy. The actual content of the nutrition and gender materials were adjusted on the basis of gaps and constraints identified in the baseline survey and feedback from the training participants.

Nutrition and gender training was planned to consist of monthly engagements with target households (alternating group‐level meetings with household‐and individual‐level coaching). However, the intervention was ultimately modified to adhere to COVID‐19‐related measures and restrictions (Figure 2). After an initial lockdown period in April and May, COVID‐19 restrictions became less stringent, though social gatherings and group trainings were still prohibited. One‐on‐one household visits with preventive measures, distribution of materials and coaching took place in June 2020, followed by additional household visits and coaching sessions in July 2020. As cases began to rise in August, renewed restrictions prevented in‐person engagements and training converted to phone‐based monitoring and coaching in October 2020.

To enable comparison between women and men on agency, decision‐making and nutrition‐related knowledge and practices, one female adult and, when possible, one male adult were selected in each household to participate in the trainings and survey. Selections were based on decision‐making in terms of livelihoods and food purchases and preparation. 3 There were no households without female adults in the sample, and 12% of sample households have only female adults.

### 2.3 Survey sample and data collection

This assessment was based on three rounds of phone surveys conducted in June, August and October 2020 (see Figure 2 and Table S1). Each round of the phone survey inquires about respondents' experiences, activities and livelihoods in the preceding months, as well as food groups consumed the day before (24‐h food recall) and frequency in eating meat, fish and vegetables in the week before the phone interview and how these indicators changed compared to the year before COVID‐19 crisis (2019). Phone surveys with village leaders of four communities were also conducted to inquire about the main COVID‐19 mitigation measures implemented in these communities and about other shocks experienced during the survey period.

The sample at baseline was 918 households, and the subsequent three phone survey rounds included 606, 543 and 503 households, respectively. Household attrition from baseline to the first round of the phone survey was 39% and was primarily driven by phone‐related issues. At baseline, 7% of households did not have a mobile phone, and an additional 7% did not provide their telephone numbers during the baseline survey. Furthermore, 25% of telephone numbers were not working during the first round. 4 Additional attrition from the first to third rounds occurred because of out‐of‐service telephone numbers, unanswered calls and interview refusals. 5 In total, household attrition was 45% from the baseline to the third round of survey.

### 2.4 Outcome indicators

The nutrition outcomes are measures of women's dietary diversity and changes in household dietary quality due to COVID‐19. The phone interviews follow the good practice recommendations on the food groupings for 24‐h recall period highlighted in FAO and FHI360 (2016). The phone interviews use a simpler list‐based format, with the respondent answering yes or no when asked whether each food group was consumed. These food groups are used to estimate women's dietary diversity score (DDS). The Minimum Dietary Diversity for Women (MDD‐W) is an internationally validated proxy indicator for the probability of micronutrient adequacy such that the population of women aged 15 to 49 years is more likely to have achieved micronutrient adequacy if on average at least 5 out of 10 healthy food groups are consumed in a 24‐h period (FAO & FHI360, 2016; Martin‐Prével et al., 2015). There is no validated cut‐off for other age groups. In this study we consider adult women primary decision‐makers, including those above 49 years, and adopt the MDD‐W threshold of five food groups as an indication of improved probability of adequate dietary diversity. The specific women's dietary diversity outcomes monitored are (1) a DDS measured by the number of healthy food groups consumed (0–10), (2) inadequate dietary diversity (<5 food groups) and (3) consumption of each food group.

A limitation is that dietary diversity captures only one aspect of dietary quality. Quantities of foods consumed may change while food groups consumed remain unvaried. To understand the impact of COVID‐19 on diets, enumerators also asked whether the household reduced the frequency (days per week) or portions of meat or fish consumed in the previous week compared to usual consumption for that time of year. Reductions in the frequency and quantity of meat or fish consumption provide additional indicators of change in dietary quality.

### 2.5 Statistical analysis

Given random assignment to the treatment, intention‐to‐treat (ITT) effects are estimated by ordinary least squares (OLS), where the variable of interest is the indicator variable equals to one if the village was assigned to the treatment group. The outcome can then be written as

Y=a+b*T+e,

where *Y* is the outcome indicator (see the previous subsection), *T* is the assignment to the treatment, *b* measures the average effect of the treatment and *a* is the constant or intercept (which can also be interpreted as the value of *Y* if *T = 0*). We test the null hypothesis (*b* = 0). If rejected, we conclude that the intervention has significant effect to the magnitude of *b*. Because the unit of randomisation is the village, standard errors from the regression models are clustered at the village level.

For the DDS, we performed a Poisson regression commonly used for count data (i.e. number of food groups consumed). As a robustness test, we also perform OLS mimicking a continuous outcome variable. For the dummy variables measuring likelihood of inadequate dietary diversity, consumption of each of the 10 food groups and reported household reduction in meat and fish consumption due to the pandemic, we performed probit models.

We took advantage of the randomised experimental design and conducted an ITT analysis using single‐difference estimation in the midline assessment data. The randomised assignment and balance in baseline characteristics minimise concerns of bias in the single‐difference treatment estimates. As a robustness check, we performed additional regressions that include various demographic controls (see Table S5 for a summary of control variables). We also performed a regression that controls for self‐reported household income loss, receipt of cash transfers and borrowing money during the COVID‐19 crisis.

Throughout our analyses, we apply inverse probability weighting to attenuate attrition bias (i.e. we computed the inverse of the probability of the attrition probit [*1/pr*] and used it as attrition weight). This procedure gives more weight to households that have similar initial characteristics as households that subsequently dropped out than to households with characteristics that make them more likely to remain in the panel.

### 2.6 Ethical considerations

This study was approved by Myanmar's Ministry of Agriculture, Livestock and Irrigation. Ethical approval for this study was obtained through the IFPRI Internal Review Board. The study is registered and reviewed by The American Economic Association's registry for randomised controlled trials. Informed oral consent was obtained from the study participants and recorded by trainers and enumerators, respectively.

## 3 RESULTS

There is no difference between those in the treatment and control villages in terms of cell phone access or in‐service telephone numbers, which indicates that the treatment effects among those with working telephone numbers and successful interviews can be extended to the full baseline sample. Attrition probit regressions were estimated to identify demographic factors that could explain the likelihood of attrition (Table S4). The characteristics of the full sample and phone survey subsample are similar, and the attrition probit shows low Pseudo R2, indicating low attrition bias.

Using a set of questions developed by Ragasa et al. (2020) to assess respondent's prior knowledge of topics covered in the LEARN modules, the baseline survey found that respondents scored particularly low on questions related to nutrition basics and family nutrition practices. Baseline assessment of respondents' food consumption in the previous 24 hours also found both men and women to be lacking the dietary diversity associated with healthy diets. Notably, the vast majority of respondents did not report consuming dairy or vitamin A‐rich fruits and vegetables (Ragasa et al., 2020). Even when beans and nuts were seemingly available in the study areas, many households did not consume them on a daily basis. At baseline, 44% of women were likely to have consumed inadequately diverse diets (<5 out of 10 food groups).

**FIGURE 3:**
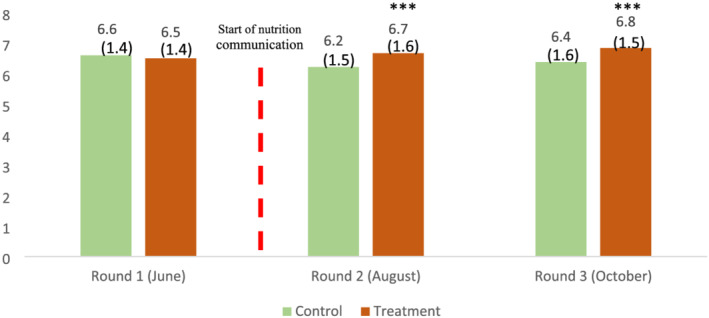
Average dietary diversity score of women, by treatment and control groups. Source: IFPRI/MSR phone surveys (June, August and October 2020). Note: Dietary diversity pertains to the 24‐h recall of food eaten the day before the phone interview in the same month as the survey. Statistical significance of the difference between control and treatment households at the 1%, 5% and 10% levels is indicated with ***, ** and *, respectively. Numbers in parenthesis are standard deviations

Figure 3 illustrates that no difference exists between treatment and control villages before the intervention (baseline and round 1 in June), whereas, once the intervention was well underway, a clear difference emerges in round 2 (August) and round 3 (October).

**FIGURE 4:**
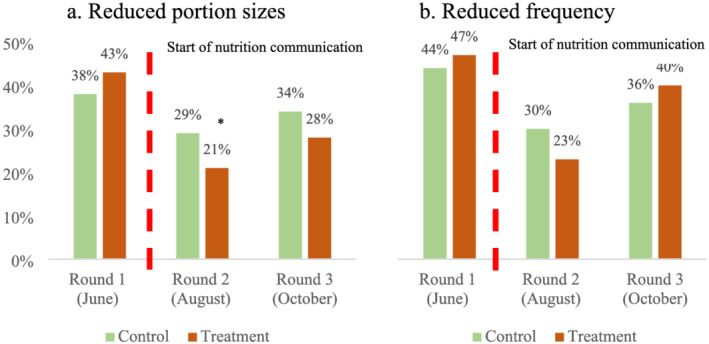
Portion of sample households reporting reduced meat or fish consumption compared to a usual year (%). Source: IFPRI/MSR phone surveys (June, August and October 2020). Note: Meat or fish consumption pertains to the 7‐day period before the phone interview. Statistical significance of the difference between control and treatment households at the 1%, 5% and 10% levels is indicated with ***, ** and *, respectively

Similarly, Figure 4 shows that, at the onset of the intervention (round 1), more households in the treatment villages reported reductions in the portion (panel a) and numbers of days per week (panel b) of meat or fish consumption. With exposure to the BCC, we immediately see a reversal: in rounds 2 and 3, fewer households in the treatment group reported reducing their meat or fish portions compared to the control group. However, the impact of the messaging on the number of days per week that meat or fish were consumed is less clear between rounds.

Table 1 presents the results of two specifications of the Poisson and probit models: (1) without controls and (2) with controls for demographics plus income loss, receipt of transfers and borrowed money dummies. In the study area, 78% and 96% of households reported receiving government cash or in‐kind transfers during the periods of June–July and August–September, respectively, compared to only 10% at baseline. We see a positive average treatment effect of the intervention on measures of women's dietary diversity and a negative effect on the likelihood of sample households reducing the quantity of meat and fish consumed during the COVID‐19 crisis compared to a usual year.

**TABLE 1:** Average treatment effect on dietary outcome indicators

Main outcomes	Treatment effect (no controls)	Treatment effect (with controls)	Control group mean	Total number of observations
	(1)	(2)	(3)	(4)
**Panel A: Poisson regression (marginal effect reported)**				
Women's dietary diversity score (0–10)^a^	0.46*** (0.17)	0.51*** (0.14)	6.296	1009
**Panel B: probit regression (marginal effect reported)**				
Likely inadequate dietary diversity among women (= 1 if score < 5)^b^	−0.06** (0.02)	−0.06*** (0.02)	0.137	1009
Food group consumption among women (= 1)^c^				
*Pulses*	0.10*** (0.04)	0.10*** (0.04)	0.700	1009
*Nuts and seeds*	0.06* (0.04)	0.08*** (0.03)	0.449	1009
*Dairy*	0.03 (0.02)	0.03 (0.02)	0.050	1009
*Meat/poultry/fish*	0.02 (0.02)	0.02 (0.02)	0.853	1009
*Eggs*	0.07 (0.05)	0.10** (0.04)	0.461	1009
*Dark green leafy vegs*.	0.01 (0.02)	0.01 (0.01)	0.932	1009
*Vitamin A‐rich fruits vegs*.	0.15** (0.07)	0.16*** (0.06)	0.431	1009
*Other vegetables*	−0.03 (0.03)	−0.02 (0.03)	0.915	1009
*Other fruits*	0.04 (0.04)	0.05 (0.04)	0.505	1009
Unusually low portions of meat/fish consumed in the household over the last 7 days (= 1)	−0.08*** (0.03)	−0.11*** (0.02)	0.318	1019
Unusually low frequency of meat/fish consumed in the household over the last 7 days (= 1)	−0.04 (0.04)	−0.07** (0.03)	0.336	1019

*Note:* Source: IFPRI/MSR phone survey (August and October 2020). All regressions use rounds 2 and 3 of the phone survey sample, with survey round fixed effects, and clustering at village. Controls in column 2 include baseline head of household characteristics (age, education level, and occupation, such as agricultural farmer, labour, or other jobs) and baseline household demographic characteristics (type of household, township, household size, dummy indicating household is a water user), and dummies indicating whether the household has income loss due to COVID‐19, has accepted transfers from government or nongovernment organisations, and has borrowed money during the COVID‐19 crisis.

^a^ Ten MDD‐W food groups.

^b^ Ten MDD‐W food groups; score < 5 indicates lower likelihood of adequate dietary diversity.

^c^ We do not report the staple food group results because all respondents report consuming staples.

* Statistical significance of coefficient estimates at the 10% level.

** Statistical significance of coefficient estimates at the 5% level.

*** Statistical significance of coefficient estimates at the 1% level.

Participation in the intervention improves women decision‐makers' DDS by about half a point compared to women in control villages (0.46 and 0.51 in specifications 1 and 2, with *p*‐value 0.008 and 0.000, SE 0.17 and 0.14, respectively). Women in treatment villages were 10% (with *p*‐values 0.007 and 0.004, SE 0.04 and 0.04, respectively) more likely to consume pulses, 6%–8% (with *p*‐values 0.073 and 0.009, SE 0.04 and 0.03, respectively) more likely to consume nuts and seeds and 15%–16% (with *p*‐values 0.027 and 0.011, SE 0.07 and 0.06, respectively) more likely to consume Vitamin A‐rich fruits and vegetables. In the specification with controls, we also see a positive effect on egg consumption. Using a cut‐off of 5 out of 10 food groups, we find 6% (*p*‐value: 0.003, SE: 0.02) fewer sample women who were likely to have consumed inadequately diverse diets.

Finally, households in treatment villages were 11% (*p*‐value: 0.000, SE: 0.02) less likely to reduce the quantity and 7% (*p*‐value: 0.020, SE: 0.07) less likely reduce the frequency of meat and fish consumption during the COVID‐19 crisis than those in control villages. However, the lower reduction in the number of days per week meat or fish that was consumed in treatment villages is significant only in the specification that controls for demographics together with changes in income.

Households in the treatment villages were 10% (*p*‐value: 0.045, SE: 0.05) more likely to report income loss than those in control villages, whereas there was no difference in receipt of transfers and borrowing money during COVID‐19 (Table S6). Controlling for self‐reported income loss, receipt of transfers and borrowing increased the average treatment effect on nearly all statistically significant outcomes (Table 1). 6

## 4 DISCUSSION

COVID‐19 policy measures had a considerable impact on livelihoods and income in the sample villages. Of the sample households, 68% reported income loss, with farming, business and nonfarm wage income impacted more heavily than farm wage income. Income loss not only hurts households directly but also has the potential compounding impact of reducing future income by diminishing funds available for production and investment (Lambrecht et al., 2020; Ragasa et al., 2020). With the implementation of the GoM's COVID‐19 Economic Relief Plan, almost all respondents in treatment and control villages received transfers (20 000 kyat per household per month) during the study period (78% in June and July and 96% between August and September). In addition, a good proportion of the respondents is applied for more loans compared to usual years. In the June–July period, 57% of households borrowed money, and 43% borrowed in the following 2 months. The borrowings were mainly regular loans (150 000 kyat/acre) and COVID‐19‐related loans (200 000 kyat/acre) from the Myanmar Agricultural Development Bank (MADB).

As income decreases, consumption of more expensive foods, such as meat and fish, is expected to fall; indeed, reduced income was the main reason cited for consuming less meat or fish by about 76% of respondents in rounds 2 and 3 of the phone survey.

Transfers and loans were expected to have eased the income effect of the pandemic. Yet income loss is so extensive that transfers and loans are most likely insufficient to fully offset losses. In the second survey round (June–July) and third survey round (August–September), 38% and 57% of households, respectively, reported that either business or other income was at least 20% lower than usual. Thus, we see most sample households reporting reduced meat and fish consumption due to income loss. Moreover, at the onset of the pandemic, 15% of respondents had false perceptions about the spread of COVID‐19 through meat or fish consumption. Those false perceptions, as well as the reported reductions in availability of protein‐rich foods such as meat and fish during periods of stringent mitigation measures, were worrying.

We demonstrate through this study that there is a role for nutrition education in complementing the financial assistance received during periods of severe shocks. Even in the presence of significant pressures to reduce consumption of more costly nutritious foods, women decision‐makers in treatment villages had greater DDS and were more likely to consume some nutrient‐dense foods (pulses, nuts and seeds, eggs, and vitamin A‐rich fruits and vegetables) than those who did not receive the nutrition and gender messaging. The treatment effect on the DDS was half a food group. Among women who received the messaging compared to those who did not, the intervention also resulted in 6% (*p*‐value: 0.003, SE: 0.02) fewer women who were likely to have inadequately diverse diets. This is an important improvement relative to the 44% of women at baseline who did not consume adequately diverse diets.

Our findings also highlight the potential for nutrition education in households with reduced income. Women living in households that reported income loss had significantly lower DDS than those who did not (Table S6). Furthermore, women in treatment households had a higher likelihood of income loss but still had higher DDS than those in the control group (Tables S6 and S8). In other words, within the treatment group, income loss did not have the negative impact on DDS that it had on those in the control group; the intervention helped mitigate the negative impact of income loss on dietary diversity.

Dietary diversity is only one aspect of dietary quality. Quantities of foods consumed within food groups can increase or decrease without changes in dietary diversity. Thus, nutrition education can affect dietary quality by encouraging households that already consume nutritious food groups to increase quantities consumed. For this reason, we look at self‐reported changes in the frequency and quantities of meat or fish consumed by households. Women in the treatment villages were not any more or less likely to have consumed meat or fish than those in control villages. However, compared to usual years, households in treatment villages were 11% (*p*‐value: 0.000, SE: 0.02) less likely to have reduced portions of meat or fish and 7% (*p*‐value: 0.020, SE: 0.03) less likely to have reduced the number of days in a week that they consumed meat or fish, compared to the control group.

Through this study, we demonstrate that nutrition and gender messaging has helped mitigate the negative supply and income impacts of the COVID‐19 crisis. We even find evidence that the messaging may have dispelled myths about the safety of meat and fish during the pandemic (Figure S1). Although impact levels may seem relatively small, they are notable midline outcomes during a period of crisis when dietary quality would be expected to deteriorate.

This study also illustrates how BCC can be effective during a crisis by combining different delivery tools to reach different types of households and individuals. Delivering nutrition education during a pandemic, such as COVID‐19, is particularly challenging with restrictions to mobility coupled with inequitable access to mobile phones and other digital media. Interventions delivered via mobile devices are among the promising approaches recently being used in urban areas and locations with high mobile phone penetration. However, in a review of 23 randomised controlled trials on healthy eating interventions delivered via mobile device, McCarroll et al. (2017) find only small positive effects on healthy eating. Furthermore, in many countries, literacy levels and access to smartphones are also lower in rural areas than in urban areas, making mobile‐based nutrition messaging more challenging.

In our study area, relying solely on mobile nutrition messaging was not possible because of the lack of phone ownership or nonfunctional phone numbers for about 39% of the baseline survey households. Moreover, the functionality of some phones was insufficient to allow users to access flyers, pictures, videos and interactive features of MIGS's mobile messaging. Adaptations to more conventional BCC techniques involved distributing materials directly to households and greater use of individual‐level coaching, both in person and via phone.

To conclude, this study shows that BCC delivered by through a range of tools, including household visits and phone‐based coaching, that are responsive to local and individual resource limitations are plausible in the setting of a pandemic. Positive effects of the BCC were already seen within 3 months of implementation on both dietary diversity and quality, as indicated by the quantity of meat and fish consumed. Nutrition messaging can complement cash transfer programmes and assist with countering declines in dietary quality that would be expected from negative shocks to supply chains and incomes. It can be a very important mechanism to support household resilience in the face of negative shocks.

## CONTRIBUTIONS

CR acted as principal investigator of the study. CR developed the study design together with IL and analysed data together with HZ. IL, KM, JS and ZWA contributed substantially on the baseline and follow‐up phone survey questionnaire and implementation. CR produced the first draft of the article. All authors were involved in the writing of the manuscript and have approved the final version for publication.

## Supporting information


**Table S1**. Survey sample time period
**Table S2.** Baseline balance check (all baseline sample)
**Table S3.** Baseline balance check (phone survey subsample only)
**Table S4.** Baseline to phone survey attrition probit analysis
**Table S5.** Summary statistics of control variables
**Table S6.** Difference in self‐reported income loss and receipt of transfer between treatment and control groups
**Table S7.** Average treatment effect on dietary outcome indicators (with weights)
**Table S8.** Mean difference between households with and without income loss by treatment status for women's minimum dietary diversity score
**Figure S1.** Proportion of sample households reporting reduced quantity or frequency of meat or fish consumption during the COVID‐19 crisis because of fears of contracting COVID‐19

## Data Availability

Data are available on request from the authors.

## Footnotes

^1^
The amount of cash transfer in the MCCT programme was 10 000 kyat/month (about USD6.50/month) and increased to 15 000 kyat/month (about USD10.00/month) since October 2017 (Field & Maffioli, 2021). The COVID‐19‐related cash transfer is 20 000 kyat/month. In comparison, the minimum daily wage is 3000 kyat. The amount of COVID‐19‐related cash transfer was equivalent to 6–7 days of work at the minimum wage.

^2^
LEARN was funded by Livelihoods and Food Security Trust Fund (LIFT) and implemented by Save the Children, Action Contre la Faim, and Helen Keller International in support of LIFT's nutrition programming in Myanmar.

^3^
This is consistent with the selection of two respondents (of opposite sex) as in the Women's Empowerment in Agriculture Index (WEAI) methodology (Malapit et al., 2019).

^4^
The GoM mandatory registration of SIM cards by 30 June 2020. This order likely led to a large share of phone numbers being blocked and users changing phone numbers. (https://www.irrawaddy.com/news/burma/myanmar-mobile-users-told-re-register-sim-cards-june-30.html).

^5^
Attrition from phone survey round 1 to round 2 was 10%. Attrition from phone survey round 1 to 3 was 17%.

^6^
Results are consistent with model estimates using attrition weights (Table S7). DDS estimates using OLS are nearly identical to those from the Poisson model and are thus not reported.
