# Supplementary material for: Can nutrition education mitigate the impacts of COVID‐19 on dietary quality? Cluster‐randomised controlled trial evidence in Myanmar's Central Dry Zone
Source: Matern Child Nutr. 2021 Aug 19;17(4):e13259. doi: 10.1111/mcn.13259 (PMC8420230; doi:10.1111/mcn.13259)
Supplement: Supplementary file 1 — Table S1. Survey sample time period Table S2. Baseline balance check (all baseline sample) Table S3. Baseline balance check (phone survey subsample only) Table S4. Baseline to phone survey attrition probit analysis Table S5. Summary statistics of control variables Table S6. Difference in self‐reported income loss and receipt of transfer between treatment and control groups Table S7. Average treatment effect on dietary outcome indicators (with weights) Table S8. Mean difference between households with and without income loss by treatment status for women's minimum dietary diversity score Figure S1. Proportion of sample households reporting reduced quantity or frequency of meat or fish consumption during the COVID‐19 crisis because of fears of contracting COVID‐19 [file MCN-17-e13259-s001.docx]

**Annex**

**Table A1. Survey sample time period**

|  | **Recall period** | **Implementation** | **Number of households** |
| --- | --- | --- | --- |
| Baseline, in-person | 2019; Jan 2020 | Jan 2020 | 918 |
| Phone survey round 1 | Feb–May 2020 | Jun 200 | 606 |
| Phone survey round 2* | June–July 2020 | Aug 2020 | 543 |
| Phone survey round 3* | Aug–Sept 2020 | Oct 2020 | 503 |

Note: * Because the intervention on gender and nutrition was implemented in June and July 2020, all analysis only uses rounds 2 and 3 of the phone survey: round 1 of the phone survey was conducted during the intervention in June and July 2020.

**Table A2. Baseline balance check (all baseline sample)**

|  | Control mean (SD) | Coefficient difference (SE) | **N** |
| --- | --- | --- | --- |
|  | (1) | (2) | (3) |
| **Panel A: Head of household** |  |  |  |
| Age | 55.300 | 1.933* | 918 |
|  | [12.438] | (1.109) |  |
| Education - did not complete primary | 0.491 | 0.004 | 918 |
|  | [0.500] | (0.059) |  |
| Education - primary school graduate | 0.430 | -0.038 | 918 |
|  | [0.496] | (0.050) |  |
| Education - at least middle school graduate | 0.079 | 0.034 | 918 |
|  | [0.270] | (0.027) |  |
| Main occupation - agricultural farmer | 0.757 | -0.050 | 918 |
|  | [0.430] | (0.034) |  |
| Main occupation - agricultural labor | 0.061 | 0.001 | 918 |
|  | [0.239] | (0.027) |  |
| Main occupation - other jobs | 0.075 | 0.018 | 918 |
|  | [0.264] | (0.018) |  |
| Main occupation - not working/homemaker | 0.108 | 0.031 | 918 |
|  | [0.301] | (0.025) |  |
| **Panel B: Household characteristics** |  |  |  |
| Household type (dual-adult households = 1) | 0.882 | 0.001 | 918 |
|  | [0.323] | (0.024) |  |
| Pale township | 0.410 | -0.037 | 918 |
|  | [0.492] | (0.185 |  |
| Tatkone township | 0.408 | -0.041 | 918 |
|  | [0.492] | (0.185) |  |
| Yinmabin township | 0.183 | 0.079 | 918 |
|  | [0.387] | (0.168) |  |
| Household size | 4.209 | 0.029 | 918 |
|  | [1.713] | (0.149) |  |
| Household is a water user | 0.817 | 0.004 | 918 |
|  | [0.387] | (0.018) |  |
| **Panel C: Survey attrition at household level** |  |  |  |
| Baseline household has phone number | 0.876 | -0.023 | 918 |
|  | [0.330] | (0.024) |  |
| Baseline household has phone number and is reachable | 0.627 | -0.039 | 918 |
|  | [0.484] | (0.058) |  |
| Total attrition between baseline and phone survey round 3 | 0.497 | -0.002 | 918 |
|  | [0.500] | (0.052) |  |

Source: IFPRI/MSR baseline survey (January 2020).

**Table A3. Baseline balance check (phone survey subsample only)**

|  | Control mean (SD) | Coefficient difference (SE) | **N** |
| --- | --- | --- | --- |
|  | (1) | (2) | (3) |
| **Panel A: Head of household** |  |  |  |
| Age | 54.798 | 1.562 | 503 |
|  | [12.314] | (1.564) |  |
| Education - did not complete primary | 0.456 | 0.019 | 503 |
|  | [0.499] | (0.074) |  |
| Education - primary school graduate | 0.440 | -0.043 | 503 |
|  | [0.497] | (0.067) |  |
| Education - at least middle school graduate | 0.105 | 0.025 | 503 |
|  | [0.307] | (0.041) |  |
| Main occupation - agricultural farmer | 0.750 | -0.048 | 503 |
|  | [0.434] | (0.041) |  |
| Main occupation - agricultural labor | 0.044 | 0.018 | 503 |
|  | [0.206] | (0.033) |  |
| Main occupation - other jobs | 0.093 | 0.009 | 503 |
|  | [0.291] | (0.028) |  |
| Main occupation - not working/homemaker | 0.113 | 0.020 | 503 |
|  | [0.317] | (0.036) |  |
| **Panel B: Household characteristics** |  |  |  |
| Household type (dual-adult household = 1) | 0.907 | -0.033 | 503 |
|  | [0.291] | (0.028) |  |
| Pale township | 0.456 | 0.003 | 503 |
|  | [0.499] | (0.190) |  |
| Tatkone township | 0.359 | 0.014 | 503 |
|  | [0.481] | (0.178) |  |
| Yinmabin township | 0.185 | -0.017 | 503 |
|  | [0.389] | (0.138) |  |
| Household size | 4.351 | -0.092 | 503 |
|  | [1.677] | (0.176) |  |
| Household is a water user | 0.819 | -0.001 | 503 |
|  | [0.386] | (0.026) |  |

Source: IFPRI/MSR baseline survey (January 2020).

**Table A4. Baseline to phone survey attrition probit analysis**

| **Explanatory Variables** | Attrition (= 1) from baseline to round 3 phone survey |
| --- | --- |
|  |  |
| Treated (= 1) | -0.012 |
|  | (0.039) |
| Age | 0.002 |
|  | (0.001) |
| Education - primary school graduate | -0.009 |
|  | (0.042) |
| Education - at least middle school graduate | -0.089 |
|  | (0.068) |
| Married | -0.048 |
|  | (0.079) |
| Widow/Widower/Divorced | 0.009 |
|  | (0.100) |
| Main occupation - Ag farmer | -0.087 |
|  | (0.085) |
| Main occupation - Other jobs | -0.165** |
|  | (0.075) |
| Main occupation - Not working/homemaker | -0.151 |
|  | (0.093) |
| Dual-headed household | 0.026 |
|  | (0.064) |
| Pale | -0.190*** |
|  | (0.065) |
| Tatkone | -0.069 |
|  | (0.070) |
| Household size | -0.014* |
|  | (0.009) |
| Household is a water user | -0.009 |
|  | (0.080) |
|  |  |
| Observations | 918 |
| Pseudo R2 | 0.0271 |
| Overall Mean | 0.496 |

Source: IFPRI/MSR baseline and phone survey.

Note: Statistical significance of coefficient estimates at the 1%, 5%, and 10% levels is indicated with ***, **, and *, respectively. Probit regression is used and marginal effect reported in the table. Attrition variable is a dummy equal to 1 if the household does not have phone number or could not be reached in round 3 of the phone survey, 0 otherwise. We got the inverse of the probability of this attrition probit (1/pr) and used it as attrition weight (see Annex Table A7).

**Table A5. Summary statistics of control variables**

|  | **Mean** | **SD** | **Min** | **Max** | **N** |
| --- | --- | --- | --- | --- | --- |
| Age (head of household) | 55.59 | 12.46 | 19 | 92 | 503 |
| **Highest education level (head of household) (= 1)** |  |  |  |  |  |
| *Did not complete primary* | 0.47 | 0.50 | 0 | 1 | 503 |
| *Primary school graduate* | 0.42 | 0.49 | 0 | 1 | 503 |
| *At least middle school graduate* | 0.12 | 0.32 | 0 | 1 | 503 |
| **Main occupation (head of household) (= 1)** |  |  |  |  |  |
| *Ag farmer* | 0.73 | 0.45 | 0 | 1 | 503 |
| *Ag labor* | 0.05 | 0.23 | 0 | 1 | 503 |
| *Other jobs* | 0.10 | 0.30 | 0 | 1 | 503 |
| *Not working/homemaker* | 0.12 | 0.33 | 0 | 1 | 503 |
| Dual-adult households (= 1) | 0.89 | 0.31 | 0 | 1 | 503 |
| **Township** |  |  |  |  |  |
| *Pale Township* | 0.46 | 0.50 | 0 | 1 | 503 |
| *Tatkone Township* | 0.37 | 0.48 | 0 | 1 | 503 |
| *Yinmabin Township* | 0.18 | 0.38 | 0 | 1 | 503 |
| Household size | 4.30 | 1.74 | 1 | 10 | 503 |
| Household is a water user (= 1) | 0.82 | 0.39 | 0 | 1 | 503 |
| **Asset quintile (= 1)** |  |  |  |  |  |
| *Asset quintile 1* | 0.19 | 0.40 | 0 | 1 | 503 |
| *Asset quintile 2* | 0.16 | 0.36 | 0 | 1 | 503 |
| *Asset quintile 3* | 0.22 | 0.42 | 0 | 1 | 503 |
| *Asset quintile 5* | 0.21 | 0.41 | 0 | 1 | 503 |
| *Asset quintile 6* | 0.21 | 0.41 | 0 | 1 | 503 |
| Total household income decreased due to COVID – PS R2 | 0.56 | 0.50 | 0 | 1 | 543 |
| Total household income decreased due to COVID – PS R3 | 0.68 | 0.47 | 0 | 1 | 503 |
| Receive and accept transfer from government OR nongovernment – PS R2 | 0.78 | 0.41 | 0 | 1 | 543 |
| Receive and accept transfer from government OR nongovernment – PS R3 | 0.96 | 0.20 | 0 | 1 | 503 |
| Borrowed money– PS R2 | 0.57 | 0.50 | 0 | 1 | 543 |
| Borrowed money– PS R3 | 0.43 | 0.50 | 0 | 1 | 503 |

Source: IFPRI/MSR phone survey (August and October 2020).

Note: PS R2 means phone survey round 2, and PS R3 means phone survey round 3.

**Table A6. Difference in self-reported income loss and receipt of transfer between treatment and control groups**

| **Outcomes** | Treatment group mean (SD) | Control group mean (SD) | Treated vs. control mean diff (SE) | Total number of observations |
| --- | --- | --- | --- | --- |
|  | (1) | (2) | (3) | (4) |
| Total household income decreased due to COVID (= 1) | 0.67 | 0.57 | 0.10** | 1,046 |
|  | (0.47) | (0.50) | (0.05) |  |
| Receive and accept transfer from government OR nongovernment organizations (= 1) | 0.87 | 0.86 | 0.01 | 1,046 |
|  | (0.34) | (0.34) | (0.06) |  |
| Borrowed money from any source (= 1) | 0.42 | 0.45 | 0.03 | 1,212 |
|  | (0.50) | (0.50) | (0.05) |  |

Source: IFPRI/MSR phone survey (August and October 2020).

Note: Statistical significance of coefficient estimates at the 1%, 5%, and 10% levels is indicated with ***, **, and *, respectively. All statistics used round 2 and round 3 of the phone survey sample and column (3) regression clusters at village.

**Table A7. Average treatment effect on dietary outcome indicators (with weights)**

| **Main Outcomes** | Treatment effect  (no controls) | Treatment effect  (with controls) | Control group mean | Total number of observations |
| --- | --- | --- | --- | --- |
|  | (1) | (2) | (3) | (4) |
| **Panel A: Poisson regression (marginal effect reported)** | | | | |
| Dietary diversity score - women (0–10) ^/a^ | 0.43** | 0.50*** | 6.296 | 1,009 |
|  | (0.17) | (0.14) |  |  |
| **Panel B: Probit regression (marginal effect reported)** | | | | |
| Likely inadequate dietary diversity among women  (= 1 if score < 5) ^/b^ | -0.05** | -0.06*** | 0.137 | 1,009 |
|  | (0.02) | (0.02) |  |  |
| Food group consumption among women (= 1) ^/c^ | | | | |
| *Pulses* | 0.10*** | 0.01*** | 0.700 | 1,009 |
|  | (0.04) | (0.04) |  |  |
| *Nuts and seeds* | 0.06* | 0.07*** | 0.449 | 1,009 |
|  | (0.03) | (0.03) |  |  |
| *Dairy* | 0.03 | 0.03 | 0.050 | 1,009 |
|  | (0.02) | (0.02) |  |  |
| *Meat/poultry/fish* | 0.02 | 0.02 | 0.853 | 1,009 |
|  | (0.02) | (0.02) |  |  |
| *Eggs* | 0.07 | 0.10** | 0.461 | 1,009 |
|  | (0.05) | (0.04) |  |  |
| *Dark green leafy vegs.* | 0.00 | 0.01 | 0.932 | 1,009 |
|  | (0.01) | (0.01) |  |  |
| *Vitamin A–rich fruits vegs.* | 0.14** | 0.16** | 0.431 | 1,009 |
|  | (0.07) | (0.06) |  |  |
| *Other vegetables* | -0.03 | -0.02 | 0.915 | 1,009 |
|  | (0.02) | (0.02) |  |  |
| *Other fruits* | 0.04 | 0.05 | 0.505 | 1,009 |
|  | (0.04) | (0.04) |  |  |
| Unusually low portions of meat/fish consumed in the household over the last 7 days (= 1) | -0.07** | -0.11*** | 0.318 | 1,019 |
|  | (0.03) | (0.02) |  |  |
| Unusually low frequency of meat/fish consumed in the household over the last 7 days (= 1) | -0.02 | -0.06** | 0.336 | 1,019 |
|  | (0.04) | (0.029) |  |  |

Source: IFPRI/MSR phone survey (August & October 2020).

Note: /a 10 MDD-W food groups; /b 10 MDD-W food groups; score <5 indicates lower likelihood of adequate dietary diversity. /c We do not report the staple food group results as all respondents report consuming staples. Statistical significance of coefficient estimates at the 1%, 5%, and 10% levels is indicated with ***, **, and *, respectively. All regressions use rounds 2 and 3 of the phone survey sample, with survey round fixed effects and clustering at village. All regressions include the inverse probability weighting of the attrition between baseline and round 3 of phone survey. Controls in column 2 include baseline head of household characteristics (age, education level, and occupation, such as agricultural farmer, labor, or other jobs) and baseline household demographic characteristics (type of household, township, household size, dummy indicating household is a water user), and dummies indicating whether the household has income loss due to COVID-19, accepted transfers from government or nongovernment organizations, or borrowed money during the COVID-19 crisis.

**Table A8. Mean difference between households with and without income loss by treatment status for women’s minimum dietary diversity score**

|  | All | Treated group mean (SD) | Control group mean (SD) | Treated vs. control mean diff (SE) |
| --- | --- | --- | --- | --- |
|  | (1) | (2) | (3) | (4) |
| With income loss | 6.45 ^/a^ | 6.69 | 6.17 | 0.52 ** |
|  | (1.56) | (1.47) | (1.61) | (0.20) |
| Without income loss | 6.65 ^/b^ | 6.88 | 6.46 | 0.42 ** |
|  | (1.55) | (1.57) | (1.52) | (0.19) |

Source: IFPRI/MSR phone survey (August & October 2020).

Note: Statistical significance of coefficient estimates at the 1%, 5%, and 10% levels is indicated with ***, **, and *, respectively. All statistics use round 2 and round 3 of the phone survey sample and column (4) regression clusters at village. Column (1) the difference between ^/a^ and ^/b^ is significant at 10% level.

**Figure A1. Proportion of sample households reporting reduced quantity or frequency of meat or fish consumption during the COVID-19 crisis because of fears of contracting COVID-19**

Source: IFPRI/MSR phone surveys (June, August and October 2020).

Note: Meat or fish consumption pertains to 7-day period before the phone interview. Statistical significance of the difference between control and treatment households at the 1%, 5%, and 10% levels is indicated with ***, **, and *, respectively.
